# Supplementary material for: Association of Genetic Polymorphisms in TLR3, TLR4, TLR7, and TLR8 with the Clinical Forms of Dengue in Patients from Veracruz, Mexico
Source: Viruses. 2020 Oct 29;12(11):1230. doi: 10.3390/v12111230 (PMC7694044; doi:10.3390/v12111230)
Supplement: Supplementary file 1 [file viruses-12-01230-s001.pdf]

viruses MDPI

Table 1. Allelic and genotypic frequencies in SNPs located in the TLR3 gene.

TLR3 SNP Genotype/allele	GP(n=46) N (%)	DF (n=100) N (%)	DHF (n=65) N (%)	DEN (DF+DHF) (n=165) N (%)	DF *vs* DHF	GP *vs* DF	GP *vs* DHF	GP *vs* DEN
**rs3775291**								
C ^{An}	122 (69)	139 (70)	95 (73)	234 (71)	1.19 (0.7 - 1.95)	4.96 (3.21 - 7.68)	5.91 (3.58 - 9.75)	5.31 (3.58 - 7.89)
T ^{Mi}	56 (31)	61 (30)	35 (27)	96 (29)				
P-value					0.48	< 0.0001	< 0.0001	< 0.0001
C/C	23 (50)	49 (49)	33 (50)	82 (50)	1.0	1.0	1.0	1.0
C/T	16 (35)	41 (41)	29 (45)	70 (42)	1.05 (0.55-2.01)	1.20 (0.56-2.57)	1.26 (0.56-2.84)	1.23 (0.60-2.50)
T/T	7 (15)	10 (10)	3 (5)	13 (8)	0.45 (0.11-1.74)	0.67 (0.23-1.99)	0.30 (0.07-1.28)	0.52 (0.19-1.46)
**P-value**					0.43	0.6	0.14	0.31
**HWE (P-value)**	0.62	0.81	0.36	0.85				
**rs6552950**								
G ^{Mi}	55 (31)	55 (28)	31 (24)	86 (26)	0.83 (0.5 - 1.37)	0.85 (0.54 - 1.32)	0.7 (0.42 - 1.171)	0.79 (0.53 - 1.18)
A ^{An}	123 (69)	145 (72)	99 (76)	244 (74)				
P-value					0.46	0.47	0.17	0.25
A/A	23 (50)	53 (53)	37 (57)	90 (55)	1.0	1.0	1.0	1.0
A/G	17 (37)	39 (39)	25 (38)	64 (39)	0.92 (0.48-1.77)	1.00 (0.47-2.11)	0.91 (0.41-2.05)	0.96 (0.48-1.95)
G/G	6 (13)	8 (8)	3 (5)	11 (6)	0.54 (0.13-2.16)	0.58 (0.18-1.86)	0.31 (0.07-1.37)	0.47 (0.16-1.40)
**P-value**					0.66	0.64	0.28	0.41
**HWE (P-value)**	0.22	0.8	1	1				

GP = General Population, DF = Febrile Dengue, DHF = Febrile Hemorrhagic Dengue, DEN = Dengue, Mi = Minor Allele, An = Ancestral Allele, HWE = Hardy Weinberg Equilibrium.

Table 2. Allelic and genotypic frequencies of TLR7 SNPs (X chromosome).

TLR7 SNP Genotype/allele	GP (n^W=59, N^M=30) N (%)	DF (n^W=51, N^M=49) N (%)	DHF (n^W=28, n^M=37) N (%)	DEN (DF+DHF) (n^W=79, n^M=86) N (%)	DF *vs* DHF	GP *vs* DF	GP *vs* DHF	GP *vs* DEN
**rs179008**								
**Men***								
T ^{Mi}	7 (23)	6 (12)	10 (27)	16 (19)	2.65 (0.87 - 8.14)	0.46 (0.13 - 1.53)	1.22 (0.39 - 3.71)	0.75 (0.27 - 2.05)
A ^{An}	23 (77)	43 (88)	27 (73)	70 (81)				
P- value					0.08	0.20	0.73	0.58
**Women**								
T ^{Mi}	28 (24)	29 (28)	19 (34)	48 (30)	1.29 (0.64 - 2.6)	1.3 (0.69 - 2.3)	(1.65 (0.82 - 3.3)	1.4 (0.81 - 2.4)
A ^{An}	90 (76)	73 (72)	37 (66)	110 (70)				
P-value					0.47	0.43	0.16	0.22
A/A	32 (54)	25 (49)	12 (43)	37 (47)	1.0	1.0	1.0	1.0
A/T	26 (44)	23 (45)	13 (46)	36 (46)	1.18 (0.45-3.10)	1.13 (0.53-2.44)	1.33 (0.52-3.41)	1.20 (0.60-2.39)
T/T	1 (2)	3 (6)	3 (11)	6 (7)	2.08 (0.36-11.89)	3.84 (0.38-	8.00 (0.76-	5.19 (0.59-

						39.19)	84.60)	45.42)
**P-value**					0.71	0.47	0.16	0.22
**HWE (P-value)**	0.15	0.73	1	0.6				
**rs3853839**								
**Men**								
G ^{Mi}	20 (67)	25 (51)	24 (65)	49 (57)	1.77 (0.73 - 4.26)	0.52 (0.2 - 1.3)	0.92 (0.33 - 2.55)	0.66 (0.27- 1.5)
C ^{An}	10 (33)	24 (49)	13 (35)	37 (43)				
P-value					0.19	0.17	0.87	0.35
**Women**								
G ^{Mi}	67 (57)	63 (62)	28 (50)	91 (58)	0.61 (0.32 - 1.19)	1.23 (0.71 - 2.11)	0.76 (0.40 - 1.44)	1.03 (0.63 - 1.67)
C ^{An}	51 (43)	39 (38)	28 (50)	67 (42)				
P-value					0.15	0.45	0.40	0.89
C/C	11 (19)	10 (20)	9 (32)	19 (24)	2.20 (0.67-7.22)	0.79 (0.27- 2.25)	1.73 (0.53- 5.65)	1.06 (0.41- 2.70)
G/C	29 (49)	19 (37)	10 (36)	29 (37)	1.29 (0.43-3.83)	0.57 (0.24- 1.32)	0.73 (0.25- 2.12)	0.61 (0.28- 1.32)
G/G	19 (32)	22 (43)	9 (32)	31 (39)	1.0	1.0	1.0	1.0
**P- value**					0.42	0.41	0.33	0.34
**HWE (P-value)**	1	0.14	0.14	0.037				

GP = General Population, DF = Dengue Fever, DHF = Dengue Hemorrhagic Fever, DEN = Dengue, Mi = Minor Allele, An = Ancestral Allele, HWE = Hardy Weinberg Equilibrium.
* TLR7-rs179008, men, allele T: 0.081 (2.654 (0.865 - 8.143)).

Table 3. Allelic and genotypic frequencies in SNPs in the TLR8 gene (X chromosome).

TLR8 SNP Genotype/allele	GP (n^W=59, n^M=30) N (%)	DF (n^W=51, N^M=49) N (%)	DHF (n^W=28, n^M=37) N (%)	DEN (DF+DHF) (n^W=79, n^M=86) N (%)	DF *vs* DHF	GP *vs* DF	GP *vs* DHF	GP *vs* DEN
**rs3764880**								
**Men**								
G $^{Mi, An}$	15 (50)	26 (53)	20 (54)	46 (53)	1.04 (0.4 - 2.45)	1.13 (0.45 - 2.80)	1.17 (0.44 - 3.08)	1.15 (0.50 - 2.64)
A	15 (50)	23 (47)	17 (46)	40 (47)				
P-value					0.92	0.79	0.74	0.74
**Women**								
G $^{Mi, An}$	71 (60)	62 (61)	31 (55)	93 (59)	0.8 (0.41 - 1.54)	1.03 (0.59 - 1.77)	0.82 (0.43 - 1.56)	0.95 (0.58 - 1.54)
A	47 (40)	40 (39)	25 (45)	65 (41)				
P-value					0.51	0.93	0.55	0.82
A/A	11 (19)	9 (18)	6 (21)	15 (19)	1.48 (0.40- 5.43)	0.94 (0.32- 2.73)	1.39 (0.40- 4.91)	1.08 (0.42- 2.80)
G/A	25 (42)	22 (43)	13 (46)	35 (44)	1.31 (0.46- 3.73)	1.01 (0.44- 2.32)	1.33 (0.48- 3.69)	1.11 (0.52- 2.35)
G/G	23 (39)	20 (39)	9 (32)	29 (37)	1.0	1.0	1.0	1.0
**P-value**					0.81	0.99	0.82	0.96
**HWE (P-value)**	0.42	0.56	0.72	0.49				
**rs5741883**								
**Men**								
T ^{Mi}	4 (13)	8 (16)	5 (14)	13 (15)	0.8 (0.24 - 2.68)	1.27 (0.35 - 4.64)	1.01 (0.25 - 4.17)	1.16 (0.35 - 3.87)
C ^{An}	26 (87)	41 (84)	32 (86)	73 (85)				
P-value					0.72	0.72	0.98	0.81
**Women**								
T ^{Mi}	16 (14)	16 (16)	7 (12)	23 (15)	0.7 (0.29 - 1.99)	1.18 (0.56 - 2.51)	0.91 (0.35 - 2.35)	1.08 (0.54 - 2.16)

C ^{An}	102 (86)	86 (84)	49 (88)	135 (85)				
P-value					0.59	0.65	0.84	0.81
C/C	45 (76)	36 (71)	21 (75)	57 (72)	1.0	1.0	1.0	1.0
C/T	12 (20)	14 (27)	7 (25)	21 (27)	0.86 (0.30-2.46)	1.46 (0.60-3.54)	1.25 (0.43-3.63)	1.38 (0.61-3.11)
T/T	2 (4)	1 (2)	0	1 (1)	0.00 (0.00-NA)	0.63 (0.05-7.17)	0.00 (0.00-NA)	0.39 (0.03-4.49)
**P-value**					0.62	0.63	0.42	0.51
**HWE (P-value)**	0.27	1	1	1				
**rs4830805**								
**Men**								
G ^{An}	18 (60)	28 (57)	18 (49)	46 (53)	0.71 (0.3 - 1.67)	0.88 (0.35 - 2.24)	0.63 (0.23 - 1.67)	0.76 (0.33 - 1.78)
A ^{Mi}	12 (40)	21 (43)	19 (51)	40 (47)				
P-value					0.43	0.80	0.35	0.53
**Women**								
G ^{An}	62 (53)	51 (50)	34 (61)	85 (54)	1.5 (0.79 - 2.99)	0.90 (0.53 - 1.53)	1.39 (0.73 - 2.66)	1.05 (0.65 - 1.69)
A ^{Mi}	56 (47)	51 (50)	22 (39)	73 (46)				
P-value					0.19	0.70	0.31	0.83
A/A	16 (27)	13 (25)	5 (18)	18 (23)	0.45 (0.12-1.68)	1.19 (0.43-3.28)	0.54 (0.15-1.88)	0.89 (0.36-2.20)
G/A	24 (41)	25 (49)	12 (43)	37 (47)	0.57 (0.20-1.63)	1.52 (0.62-3.75)	0.86 (0.31-2.38)	1.22 (0.55-2.69)
G/G	19 (32)	13 (26)	11 (39)	24 (3)	1.0	1.0	1.0	1.0
**P-value**					0.42	0.64	0.61	0.75
**HWE (P-value)**	0.19	1	0.69	0.65				
**rs1548731**								
**Men**								
C	24 (80)	40 (82)	30 (81)	70 (81)	0.96 (0.32 - 2.8)	0.9 (0.28 - 2.84)	0.93 (0.27 - 3.14)	0.91 (0.32 - 2.60)
T $^{Mi, An}$	6 (20)	9 (18)	7 (19)	16 (19)				
P-value					0.44	0.85	0.91	0.86
**Women**								
C	97 (82)	86 (84)	47 (84)	133 (84)	1.02 (0.42 - 2.5)	0.86 (0.42 - 1.75)	0.88 (0.37 - 2.08)	0.86 (0.46 - 1.64)
T $^{Mi, An}$	21 (18)	16 (16)	9 (16)	25 (16)				
P-value					0.94	0.67	0.77	0.66
C/C	42 (71)	36 (71)	19 (68)	55 (70)	1.0	1.0	1.0	1.0
C/T	13 (22)	14 (27)	9 (32)	23 (29)	1.22 (0.45-3.33)	1.26 (0.52-3.02)	1.53 (0.56-4.19)	1.35 (0.61-2.98)
T/T	4 (7)	1 (2)	0	1 (1)	0.00 (0.00-NA)	0.29 (0.03-2.73)	0.00 (0.00-NA)	0.19 (0.02-1.77)
**P- value**					0.6	0.4	0.14	0.17
**HWE (P-value)**	0.068	0.73	1	0.68				

GP = General Population, DF = Dengue Fever, DHF = Dengue Hemorrhagic Fever, DEN = Dengue, W=Women, M=Men, Mi = Minor Allele, An = Ancestral Allele, HWE = Hardy Weinberg Equilibrium.

Table 4. Allelic and genotypic frequencies in SNPs in the TLR8 gene (X chromosome).

**TLR8 SNP**	**GP (n^W=59,**	**DF**	**DHF (n^W=28,**	**DEN (DF+DHF)**	**DF*vs* DHF**	**GP*vs* DF**	**GP*vs* DHF**	**GP*vs* DEN**

Genotype/allele	n^{M}=30) N (%)	(n^{W}=51, n^{M}=49) N (%)	n^{M}=37) N (%)	(n^{W}=79, n^{M}=86) N (%)				
**rs4830805**								
**Men**								
G ^{An}	18 (60)	28 (57)	18 (49)	46 (53)	0.71 (0.3 - 1.67)	0.88 (0.35 - 2.24)	0.63 (0.23 - 1.67)	0.76 (0.33 - 1.78)
A ^{Mi}	12 (40)	21 (43)	19 (51)	40 (47)				
P-value					0.43	0.80	0.35	0.53
**Women**								
G ^{An}	62 (53)	51 (50)	34 (61)	85 (54)	1.5 (0.79 - 2.99)	0.90 (0.53 - 1.53)	1.39 (0.73 - 2.66)	1.05 (0.65 - 1.69)
A ^{Mi}	56 (47)	51 (50)	22 (39)	73 (46)				
P-value					0.19	0.70	0.31	0.83
A/A	16 (27)	13 (25)	5 (18)	18 (23)	0.45 (0.12-1.68)	1.19 (0.43-3.28)	0.54 (0.15-1.88)	0.89 (0.36-2.20)
G/A	24 (41)	25 (49)	12 (43)	37 (47)	0.57 (0.20-1.63)	1.52 (0.62-3.75)	0.86 (0.31-2.38)	1.22 (0.55-2.69)
G/G	19 (32)	13 (26)	11 (39)	24 (3)	1.0	1.0	1.0	1.0
**P-value**					0.42	0.64	0.61	0.75
**HWE (P-value)**	0.19	1	0.69	0.65				
**rs1548731**								
**Men**								
C	24 (80)	40 (82)	30 (81)	70 (81)	0.96 (0.32 - 2.8)	0.9 (0.28 - 2.84)	0.93 (0.27 - 3.14)	0.91 (0.32 - 2.60)
T $^{Mi, An}$	6 (20)	9 (18)	7 (19)	16 (19)				
P-value					0.44	0.85	0.91	0.86
**Women**								
C	97 (82)	86 (84)	47 (84)	133 (84)	1.02 (0.42 - 2.5)	0.86 (0.42 - 1.75)	0.88 (0.37 - 2.08)	0.86 (0.46 - 1.64)
T $^{Mi, An}$	21 (18)	16 (16)	9 (16)	25 (16)				
P-value					0.94	0.67	0.77	0.66
C/C	42 (71)	36 (71)	19 (68)	55 (70)	1.0	1.0	1.0	1.0
C/T	13 (22)	14 (27)	9 (32)	23 (29)	1.22 (0.45-3.33)	1.26 (0.52-3.02)	1.53 (0.56-4.19)	1.35 (0.61-2.98)
T/T	4 (7)	1 (2)	0	1 (1)	0.00 (0.00-NA)	0.29 (0.03-2.73)	0.00 (0.00-NA)	0.19 (0.02-1.77)
**P- value**					0.6	0.4	0.14	0.17
**HWE (P-value)**	0.068	0.73	1	0.68				

GP = General Population, DF = Dengue Fever, DHF = Dengue Hemorrhagic Fever, DEN = Dengue, W=Women, M=Men, Mi = Minor Allele, An = Ancestral Allele, HWE = Hardy Weinberg Equilibrium.

Table 5. Allelic and genotypic frequencies of TLR4 SNPs.

TLR4 SNP Genotype/allele	GP (n=89) N (%)	DF (n=100) N (%)	DHF (n=65) N (%)	DEN (DF+DHF) (n=165) N (%)	DF *vs* DHF	GP *vs* DF	GP *vs* DHF	GP *vs* DEN
**rs4986790**								
G^{Mi}	5 (3)	8 (4)	8 (6)	16 (5)	1.57 (0.57 - 4.3)	1.44 (0.46 – 4.49)	2.26 (0.72 – 7.1)	1.76 (0.63 – 4.89)
A^{An}	173 (97)	192 (96)	122 (94)	314 (95)				
P-value					0.37	0.52	0.15	0.27

A/A	86 (97)	94 (94)	61 (94)	155 (94)	1.0	1.0	1.0	1.0
A/G	1(1)	4 (4)	0	4 (2)	-	3.66 (0.4-33.5)	-	2.22 (0.24-20.17)
G/G	2 (2)	2 (2)	4 (6)	6 (4)	3.08 (0.55-17.34)	0.91 (0.13-6.64)	2.82 (0.50-15.89)	1.66 (0.33-8.43)
**P-value**					0.054	0.44	0.27	0.62
**HWE (P-value)**	0.0005	0.0052	< 0.0001	<0.0001				
**rs4986791**								
T[Mi]	2 (1)	4 (2)	2 (2)	6 (2)	1.3(0.23 - 7.23)	1.79 (0.32 - 9.92)	1.37 (0.19 – 9.89)	1.63 (0.32 – 8.16)
C[An]	176 (99)	196 (98)	128 (98)	324 (98)				
P-value					0.76	0.49	0.75	0.54
C/C	87 (98)	96 (96)	63 (97)	159 (96)	1.0	1.0	1.0	1.0
C/T	2 (2)	4 (4)	2 (3)	6 (4)	0.76 (0.14-4.28)	1.81 (0.32-10.14)	1.38 (0.19-10.07)	1.64 (0.32-8.31)
**P-value**					0.75	0.49	0.75	0.53
**HWE (P-value)**	1	1	1	1				
**rs10759932**								
T [An]	162 (91)	178 (89)	118 (91)	296 (90)	1.22 (0.58 - 2.55)	0.79 (0.40 – 1.57)	0.97 (0.44 – 2.13)	0.85 (0.46 – 1.6)
C [Mi]	16 (9)	22 (11)	12 (9)	34 (10)				
P-value					0.61	0.51	0.94	0.63
C/C	1 (1)	1 (1)	0	1 (1)	0.00 (0.00-NA)	0.94 (0.06-15.25)	-	0.56 (0.03-9.09)
T/C	14 (16)	20 (20)	12 (18)	32 (19)	0.89 (0.40-1.98)	1.34 (0.63-2.84)	1.20 (0.51-2.79)	1.28 (0.64-2.55)
T/T	74 (83)	79 (79)	53 (82)	132 (80)	1.0	1.0	1.0	1.0
P-value					0.58	0.75	0.53	0.71
**HWE (P-value)**	0.52	1	1	1				
**rs10983755**								
G [An]	172 (97)	197 (98)	130 (100)	327 (99)	4.62 (0.23 - 90.3)	2.29 (0.56 – 9.3)	9.83 (0.54 - 176)	3.8 (0.93 – 15.4)
A [Mi]	6 (3)	3 (2)	0	3 (1)				
P-value					0.16	0.23	0.041	0.044
A/A	3 (3)	1 (1)	0	1 (1)	-	0.29 (0.03-2.86)	-	0.18 (0.02-1.72)
G/A	0	1 (1)	0	1 (1)	-	-	-	-
G/G	86 (97)	98 (98)	100	163 (98)	1.0	1.0	1.0	1.0
**P-value**					0.36	0.31	0.068	0.17
**HWE (P-value)**	<0.0001	0.015	1	0.0091				

GP = General Population, DF = Dengue Fever, DHF = Dengue Hemorrhagic Fever, DEN = Dengue, W=Women, M=Men, Mi = Minor Allele, An = Ancestral Allele, HWE = Hardy Weinberg Equilibrium.
